# Hypoxia-induced carbonic anhydrase IX facilitates lactate flux in human breast cancer cells by non-catalytic function

**DOI:** 10.1038/srep13605

**Published:** 2015-09-04

**Authors:** Somayeh Jamali, Michael Klier, Samantha Ames, L. Felipe Barros, Robert McKenna, Joachim W. Deitmer, Holger M. Becker

**Affiliations:** 1 Division of Zoology/Membrane Transport, FB Biologie, TU Kaiserslautern, P.O. Box 3049, D-67653 Kaiserslautern, Germany; 2 Division of General Zoology, FB Biologie, TU Kaiserslautern, P.O. Box 3049, D-67653 Kaiserslautern, Germany; 3 Centro de Estudios Científicos (CECs), Valdivia, Chile; 4 Department of Biochemistry and Molecular Biology, University of Florida, Gainesville, FL 32610, USA

## Abstract

The most aggressive tumour cells, which often reside in hypoxic environments, rely on glycolysis for energy production. Thereby they release vast amounts of lactate and protons via monocarboxylate transporters (MCTs), which exacerbates extracellular acidification and supports the formation of a hostile environment. We have studied the mechanisms of regulated lactate transport in MCF-7 human breast cancer cells. Under hypoxia, expression of MCT1 and MCT4 remained unchanged, while expression of carbonic anhydrase IX (CAIX) was greatly enhanced. Our results show that CAIX augments MCT1 transport activity by a non-catalytic interaction. Mutation studies in *Xenopus* oocytes indicate that CAIX, via its intramolecular H^+^-shuttle His200, functions as a “proton-collecting/distributing antenna” to facilitate rapid lactate flux via MCT1. Knockdown of CAIX significantly reduced proliferation of cancer cells, suggesting that rapid efflux of lactate and H^+^, as enhanced by CAIX, contributes to cancer cell survival under hypoxic conditions.

The most aggressive and invasive tumour cells, which often reside in hypoxic environments, rely on extensive glycolysis to meet their large demand for energy and biosynthetic precursors^1^,^2^,^3^,^4^. The excessive glycolytic activity is often triggered by hypoxia, which derives from high cell density, accompanied by insufficient vascularization^5^,^6^,^7^. However, up-regulation of glycolysis can also be observed in cancer cells under aerobic conditions, a phenomenon termed ‘Warburg effect’^8^,^9^. The increase in glycolysis leads to vast production of lactate and protons, which have to be removed from the cell to prevent acidosis, which, among other effects, would result in inhibition of glycolysis.

Efflux of lactate from cancer cells is primarily mediated by the monocarboxylate transporters MCT1 and MCT4, both of which carry lactate in co-transport with H^+^^7^,^10^,^11^,^12^. MCT-mediated H^+^ efflux exacerbates extracellular acidification and supports the formation of a hostile environment which favours tumour growth^3^,^7^,^13^,^14^,^15^. This acidic environment is created by the tumour cell-specific activation of pH regulatory mechanisms (predominantly by activation of the Na^+^/H^+^ exchanger NHE1 and in some cases the Na^+^/HCO_3_^−^ cotransporter NBCn1) which results in alkaline cytosol and acidic extracellular space^3^,^6^,^16^,^17^,^18^,^19^,^20^. Low extracellular pH, which can drop to values well below 6.5, together with local hypoxia creates a hostile environment, in which cancer cells, specifically adapted to these conditions, easily outcompete normal cells, which further enhances continued tumour progression^13^. Furthermore, these changes in the microenvironment allow tumour cells to escape conventional anti-cancer therapies^13^.

Another key protein in tumour acid/base regulation is the hypoxia-regulated, membrane-tethered, extracellular carbonic anhydrase CAIX, which catalyses the reversible hydration of CO_2_ to HCO_3_^−^ + H^+^. CAIX, the expression of which is usually linked to poor prognosis, drives HCO_3_^−^ import via Na^+^/HCO_3_^−^ cotransporters (NBCs) and Cl^−^/HCO_3_^−^ exchangers (AEs) and facilitates CO_2_ diffusion, leading to exacerbated intracellular alkalization and extracellular acidification^20^,^21^,^22^,^23^,^24^. Furthermore CAIX might function as a pro-migratory factor which facilitates cell movement and invasion^20^,^24^,^25^,^26^.

While the hostile tumour environment represents an obstacle for conventional anti-tumour agents, the alterations in cell metabolism and pH regulation might present auspicious targets for new tumour therapies. Especially MCT1, MCT4 and CAIX provide tenderizing targets to initiate cancer cell-specific ‘suicide’^3^,^15^. While inhibitors of CAIX are currently in clinical trials^27^, increasing effort is put into the detailed analysis of the manifold functions of MCTs and CAs in tumour metabolism and acid/base regulation that might provide new angles for innovative cancer therapies. For detailed reviews on energy metabolism and pH dynamics in tumours see^2^,^3^,^4^,^7^,^19^,^20^,^23^.

In the present study we used the human breast cancer cell lines MCF-7 and MDA-MB-231 as model systems to study regulation of lactate flux in cancer cells under normoxic and hypoxic conditions. The experiments revealed that cancer cells increase lactate production and lactate transport capacity under hypoxic conditions. Interestingly, lactate flux was *not* augmented by increased expression of MCTs, but by hypoxia-induced upregulation of CAIX, which greatly enhances lactate transport. Knockdown of CAIX led to a significant reduction in cell proliferation which was almost as efficient as complete pharmacological inhibition of lactate efflux. Therefore, the non-catalytic interaction between MCTs and CAIX in hypoxic cancer cells could provide a new therapeutic target that would not be exploited by common inhibitors that only target CAIX catalytic activity.

## Results

### Hypoxia-induced CAIX facilitates lactate/H^+^ flux by non-catalytic interaction

**Figure 1:**
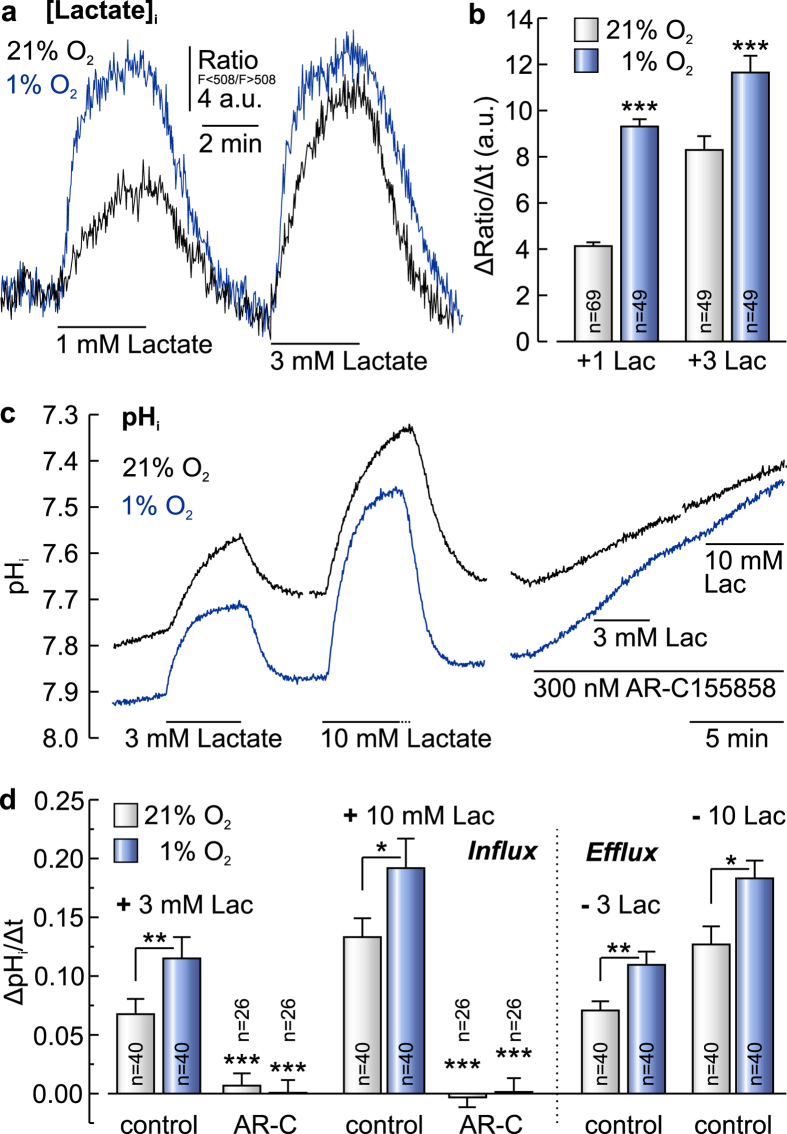
Lactate flux in MCF-7 cancer cells is augmented under hypoxic conditions. (**a**) Relative change in intracellular lactate concentration in MCF-7 cells under normoxic (21% O_2_, black trace) and hypoxic (1% O_2_, blue trace) conditions, respectively, as induced by application of 1 and 3 mM lactate, measured with *Laconic*. (**b**) Rate of change in intracellular lactate concentration in MCF-7 cells under normoxic (21% O_2_) and hypoxic (1% O_2_) conditions, respectively, as induced by application of 1 and 3 mM lactate. Hypoxia induces a significant increase in lactate flux. (**c**) Change in intracellular pH (pH_i_) in MCF-7 cells under normoxic (21% O_2_, black trace) and hypoxic (1% O_2_, blue trace) conditions, respectively, as induced by application of 3 and 10 mM lactate in the absence and presence of the MCT1 inhibitor AR-C155858. (**d**) Rate of change in pH_i_ in MCF-7 cells under normoxic (21% O_2_) and hypoxic (1% O_2_) conditions, respectively, as induced by application and removal, of 3 and 10 mM lactate, respectively. Rate of lactate-induced proton flux is augmented under hypoxic conditions. AR-C155858 fully inhibits proton flux. Data are represented as mean ± SEM.

Hypoxia triggers a glycolytic switch in tumour tissues, resulting in increased production of lactate and H^+^. To test whether lactate/H^+^ transport capacity is increased by hypoxia, we measured lactate flux in MCF-7 cells during application of 1 and 3 mM lactate under normoxic and hypoxic conditions by single-cell lactate imaging with the FRET-based lactate nanosensor *Laconic* (Fig. 1a). Indeed, the rate of lactate rise increased to 225% at 1 mM and 140% at 3 mM lactate under hypoxic conditions (Fig. 1b). Since *Laconic* has a high affinity for lactate, the sensor is well suited to reliably measure the rate of lactate uptake, but not the rate of lactate efflux (due to the high time constant of lactate release from the sensor the rate of efflux may be underestimated). To determine efflux capacity, we measured changes in intracellular pH in MCF-7 cells during application and removal of 3 and 10 mM lactate under normoxic and hypoxic conditions by pH imaging (Fig. 1c). Under hypoxia, the lactate-induced rate of change in pH_i_ increased both during application and removal of lactate, suggesting a hypoxia-dependent increase in lactate/H^+^ influx and efflux (Fig. 1d). Application of 3 and 10 mM lactate in the presence of the MCT1 inhibitor AR-C155858 (300 nM) led to no change in pH_i_ neither in normoxic, nor in hypoxic cells (Fig. 1c,d). Since the cells constantly produce lactate and H^+^, inhibition of lactate/H^+^ efflux leads to a constant intracellular acidification (Fig. 1c). Therefore ΔpH_i_/Δt measured shortly after application of AR-C155858 was subtracted from ΔpH_i_/Δt measured during application of lactate in the presence of the inhibitor.

**Figure 2:**
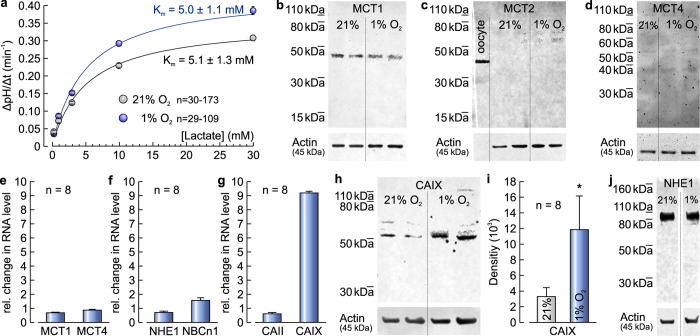
Expression of CAIX but not of MCT1 and MCT4 is upregulated under hypoxic conditions. (**a**) Determination of the K_m_ value for lactate in MCF-7 cells under normoxic (21% O_2_, grey) and hypoxic (1% O_2_, blue) conditions, respectively, as determined by the rate of change in pH_i_ during application of 0.3, 1, 3, 10 and 30 mM lactate. Western blots of lysate from MCF-7 cells, incubated under normoxic (21% O_2_) and hypoxic (1% O_2_) conditions, labelled for MCT1 (**b**), MCT2 (**c**) and MCT4 (**d**), respectively. For positive control of MCT2, lysate from MCT2-expressing oocytes was used. Actin was used as loading control. (**e**) Relative change in the RNA level of MCT1 and MCT4 in MCF-7 cells after three days under hypoxic conditions. (**f**) Relative change in the RNA level of NHE1 and NBCn1 in MCF-7 cells after three days under hypoxic conditions. (**g**) Relative change in the RNA level of CAII and CAIX in MCF-7 cells after three days under hypoxic conditions. Expression level of CAIX is strongly upregulated, while the expression levels of MCT1, MCT4, NHE1 and NBCe1 show no significant changes. (**h**) Western blot of MCF-7 cell lysate, labelled for CAIX and actin as loading control. (**i**) Quantification of CAIX protein level by western blot analysis in MCF-7 cells under normoxic (21% O_2_) and hypoxic (1% O_2_) conditions, respectively. (**j**) Western blots of lysate from MCF-7 cells, incubated under normoxic (21% O_2_) and hypoxic (1% O_2_) conditions, labelled for NHE1 and actin. Data are represented as mean ± SEM.

To analyse which MCT isoforms mediate lactate/H^+^ flux under normoxic and hypoxic conditions, we determined the K_m_ value for lactate in MCF-7 cells under both conditions by measuring the rate of change in pH_i_ during application of different lactate concentrations (Fig. 2a). Both, under normoxia and hypoxia, the K_m_ value was ~5 mM. For MCT1 a K_m_ value between 3–8 mM had been determined in various cell types^28^,^29^,^30^. For MCT2 the K_m_ value was found to be 0.74^31^, while for MCT4 K_m_ values between 17–35 mM have been reported^32^. Together with the total inhibition of lactate-induced acidification with AR-C155858, these results lead to the conclusion that lactate flux in MCF-7 cells is exclusively mediated by MCT1. This conclusion is further supported by western blot analysis for MCT1, MCT2 and MCT4 (Fig. 2b–d). Only for MCT1 sharp bands could be observed for MCF-7 cells both under normoxic and hypoxic conditions (Fig. 2b). For MCT2 no bands could be observed in MCF-7 cells under either condition, while cell lysate from MCT2-expressing *Xenopus* oocytes, used as positive control, produced a single sharp band at ~40 kDa (Fig. 2c). For MCT4 only faint bands where visible in MCF-7 cells under normoxia or hypoxia (Fig. 1d), while the same antibody produced robust bands in MDA-MB-231 cells (Fig. S1d).

Lactate/H^+^ cotransport in cancer cells has been reported to be primarily mediated by the monocarboxylate transporters MCT1 and MCT4, expression of the latter being under control of the hypoxia-inducible factor HIF1α^3^. Interestingly, neither expression of MCT1 nor MCT4 was increased under hypoxic conditions, as determined by relative quantification of mRNA levels by RT-qPCR (Fig. 2e) and by quantification of protein levels by western blot (Fig. 2b,d). Therefore, the increase in lactate transport capacity during hypoxia is most likely *not* mediated by a change in the expression levels of monocarboxylate transporters. Since MCTs cotransport lactate with H^+^, transport activity is linked to the cellular acid/base regulatory mechanisms. Therefore we investigated possible effects of hypoxia on the expression level of the main acid/base-regulating proteins found in MCF-7 cells (NHE1, NBCn1, CAII and CAIX) by RT-qPCR. Of all genes studied, only CAIX showed a strong upregulation, while only small changes in the expression level of NHE1, NBCn1 and CAII were observed (Fig. 2f,g,j). The robust upregulation of CAIX was confirmed at the protein level by western blot analysis (Fig. 2h,i). To confirm that the observed hypoxia-dependent changes in the expression pattern of these acid/base-regulatory proteins are not specific to the MCF-7 cell line, we also determined the expression levels of MCT1, MCT4, NHE1, NBCn1, CAII, and CAIX in the triple negative breast cancer cell line MDA-MB-231. Hypoxia induced a slight increase in the RNA levels of MCT1 and MCT4 (Fig. S1a), however no increase in MCT1/4 protein content was observed under hypoxic conditions (Fig. S1d,e). Like in MCF-7 cells, hypoxia led to a strong upregulation of CAIX, both on the RNA and protein level (Fig. S1c,d,f), while only moderate changes in the RNA levels of the other acid/base regulators NHE1, NBCn1 and CAII occurred under these conditions (Fig. S1b,c).

**Figure 3:**
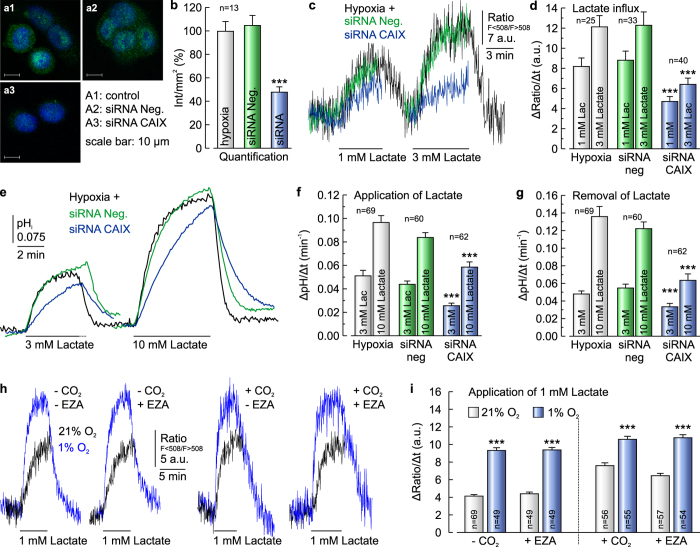
Knockdown of CAIX decreases lactate transport in cancer cells. (**a**) Antibody staining for CAIX (green) in MCF-7 cells, kept under hypoxic conditions. Hypoxic cells either remained untreated (a1), mock-transfected with non-targeting negative control siRNA (a2) or transfected with siRNA against CAIX (a3). Nuclei are stained with Hoechst (blue). (**b**) Quantification of the fluorescent signal for CAIX as shown in (a). (**c**) Original recording of the relative change in intracellular lactate concentration in MCF-7 cells kept under hypoxic conditions during application of 1 and 3 mM lactate. Cells were either untreated (black trace), mock-transfected with non-targeting negative control siRNA (green trace) or transfected with siRNA against CAIX (blue trace). (**d**) Rate of change in lactate level during application of 1 and 3 mM lactate in hypoxic MCF-7 cells, either untreated (gray bars), mock-transfected with non-targeting negative control siRNA (green bars) or transfected with siRNA against CAIX (blue bars). Knock-down of CAIX induced a significant decrease in lactate flux. (**e**) Original recordings of changes in pH_i_ in hypoxic MCF-7 cells, either untreated (control, gray traces), mock-transfected with non-targeting negative control siRNA (green traces) or transfected with siRNA against CAIX (blue traces). (**f**,**g**) Rate of change in pH_i_, as induced by application (**f**) and removal (**g**) of lactate, respectively. (**h**) Original recordings of the relative change in intracellular lactate concentration in MCF-7 cells kept under normoxic (blue traces) or hypoxic (red traces) conditions during application of lactate in the presence and absence of 5% CO_2_/15 mM HCO_3_^−^ and 30 μM EZA, respectively. (**i**) Rate of change in intracellular lactate concentration in MCF-7 cells under normoxic and hypoxic conditions, respectively, as induced by application of 1 mM lactate in the absence and presence of 5% CO_2_/15 mM HCO_3_^−^ and 30 μM EZA, respectively. Hypoxia induces a significant increase in lactate flux both in the absence and in the presence of CO_2_/HCO_3_^−^ and EZA. Knockdown of CAIX induced a significant decrease in the rate of change in pH_i_, both during addition and removal of lactate. Data are represented as mean ± SEM.

To determine whether lactate transport is augmented by CAIX, we knocked down CAIX in hypoxic MCF-7 cells (Fig. 3). Transfection of cells with siRNA against CAIX reduced expression of CAIX to 46%, while transfection of cells with non-targeting negative control siRNA had no effect on CAIX expression level (Fig. 3a,b). The rate of lactate flux, as measured with *Laconic*, decreased to ~50% when CAIX was knocked down in hypoxic MCF-7 cells (Fig. 3c,d). These data fit well to the doubling in lactate transport in hypoxic cells as compared to normoxic cells (see Fig. 1b). Like lactate flux, the rate of change in pH_i_ (ΔpH_i_/Δt), as induced by application and removal of lactate, also decreased after knockdown of CAIX under hypoxic conditions in MCF-7 cells, while transfection with control siRNA had no significant effect on ΔpH_i_/Δt (Fig. 3e–g).

Carbonic anhydrases catalyse the reversible hydration of CO_2_ to H^+^ and HCO_3_^−^ and could therefore provide substrate (H^+^) for MCT transport activity. However, inhibition of CA catalytic activity with 30 μM 6-ethoxy-2-benzothiazolesulfonamide (EZA) had no influence on the hypoxia-dependent augmentation in lactate flux, neither in the presence nor in the absence of 5% CO_2_/15 mM HCO_3_^−^ (Fig. 3h,i, S2a). This observation could be confirmed by measurement of the rate of change in pH_i_ during application and removal of lactate: The hypoxia-dependent augmentation in ΔpH_i_/Δt during application and removal of lactate was not altered by inhibition of CA catalytic activity with 30 μM EZA, both in the absence and presence of CO_2_/HCO_3_^−^ (Fig. S2b,c). Measurements on *Xenopus* oocytes confirmed that 30 μM EZA is sufficient to fully inhibit CAIX catalytic activity (Fig. S3a,c). These data suggest that the increase in lactate/H^+^ flux under hypoxic conditions is mediated by a non-catalytic function of CAIX.

### CAIX functions as a ‘proton collecting/distributing antenna’

**Figure 4:**
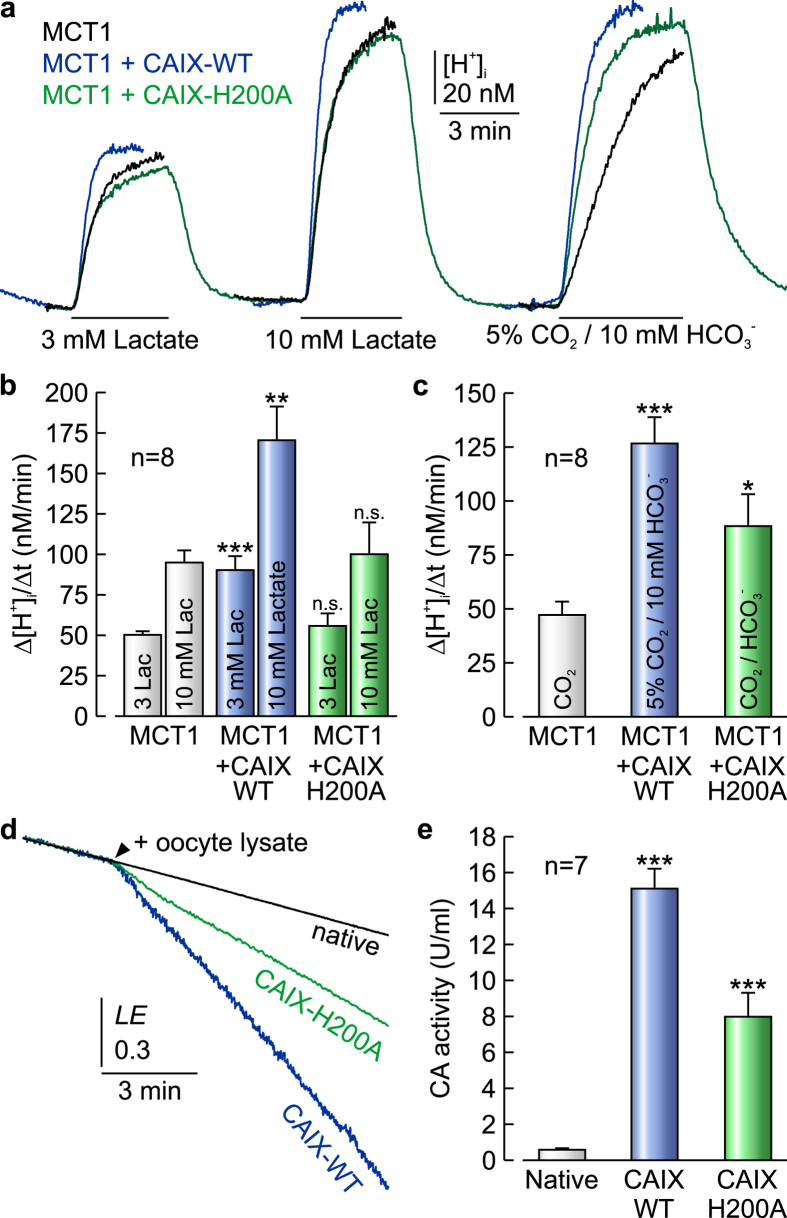
CAIX enhances MCT transport activity by facilitating its intramolecular H^+^ shuttle. (**a**) Original recordings of intracellular H^+^-concentration ([H^+^]_i_) in *Xenopus* oocytes expressing MCT1 (black trace), MCT1+CAIX-WT (blue trace), or MCT1+CAIX-H200A (green trace), respectively, during application of 3 and 10 mM lactate and of 5% CO_2_/10 mM HCO_3_^−^. (**b**,**c**) Rate of rise in [H^+^]_i_, as induced by application of lactate (**b**) or 5% CO_2_/10 mM HCO_3_^−^ (**c**) in oocytes expressing MCT1, MCT1+CAIX-WT and MCT1+CAIX-H200A, respectively. (**d**) Original recordings of the log enrichment of 20 native oocytes and 20 oocytes expressing either CAIX-WT or CAIX-H200A. The beginning of the traces shows the rate of degradation of the ^18^O-labeled substrate in the non-catalysed reaction. The black arrowhead indicates addition of oocytes. (**e**) Enzymatic activity of native oocytes and oocytes expressing either CAIX-WT or CAIX-H200A. One unit is defined as 100% stimulation of the non-catalysed ^18^O depletion of doubly labelled ^13^C^18^O_2_. Data are represented as mean ± SEM.

CAIX facilitates an intramolecular H^+^ shuttle, with the histidine at position 200, to move H^+^ between its catalytic centre and the surrounding bulk solution. To test whether CAIX augments MCT1 transport activity by facilitating this H^+^ shuttle, we replaced His200 by alanine (CAIX-H200A) and coexpressed either CAIX-WT or the mutant CAIX-H200A with MCT1 in *Xenopus* oocytes (Fig. 4a). Coexpression of MCT1 with CAIX-WT increased Δ[H^+^]_i_/Δt to 180% (Fig. 4b), a value quite similar to the augmentation in lactate transport observed in MCF-7 cancer cells. Like in MCF-7 cells, the CAIX-mediated increase in MCT1 transport activity in *Xenopus* oocytes was independent from CAIX catalytic activity, as indicated by the persistence of the increase in Δ[H^+^]_i_/Δt in the presence of 30 μM EZA (Fig. S3a,b). Furthermore, CAIX did not increase the expression level of MCT1, as evaluated by western blot analysis (Fig. S3d). However, coexpression of MCT1 with CAIX-H200A did not increase the rate of lactate-induced acidification (Fig. 4a,b). Compared to CAIX-WT, catalytic activity of CAIX-H200A decreased to 70% and 53%, when determined by pH_i_ measurement during application of CO_2_/HCO_3_^−^ (Fig. 4a,c) and by gas analysis mass spectrometry, respectively (Fig. 4d,e). This reduction in catalytic activity corresponds to the reduction in activity determined for CAII-H64A (51%), the equivalent to CAIX-H200A^33^. The failure of CAIX-H200A to enhance MCT1 transport activity suggests that CAIX functions as a ‘proton-collecting/distributing antenna’ which facilitates its intramolecular H^+^ shuttle to transfer protons between transporter pore and surrounding protonatable residues. This interaction with MCT1 would require that CAIX is located at the extracellular surface of the plasma membrane. To confirm that MCT1 transport activity is *not* mediated by *intracellular* CAIX, we injected 20 ng of purified CAIX protein (which is trapped inside the cytosol) into MCT1-expressing oocytes (Fig. S3a–c). Injection of CAIX protein displayed intracellular catalytic activity, as observed from the increased rate of acidification during application of CO_2_/HCO_3_^−^, but had no effect on MCT1 transport activity. This indicates that the augmentation of transport activity in MCT1+CAIX-coexpressing oocytes is mediated by extracellular CAIX.

While lactate transport in MCF-7 cells is mediated by MCT1 alone, expression of MCT4 has been shown in different other cancer cells, especially under hypoxic conditions^3^. Therefore we investigated in the oocyte model whether transport activity of MCT4 is also enhanced by CAIX. Indeed, analysis of MCT4 transport activity in *Xenopus* oocytes coexpressing MCT4 and CAIX revealed that CAIX facilitates MCT4 activity to a similar extent as shown for MCT1 (Fig. S3e–g). Therefore we hypothesize that hypoxia-regulated CAIX might also augment lactate efflux in cancer cells that primarily express MCT4 under hypoxic conditions.

### MCF-7 cells switch to glycolytic energy production under hypoxic conditions

**Figure 5:**
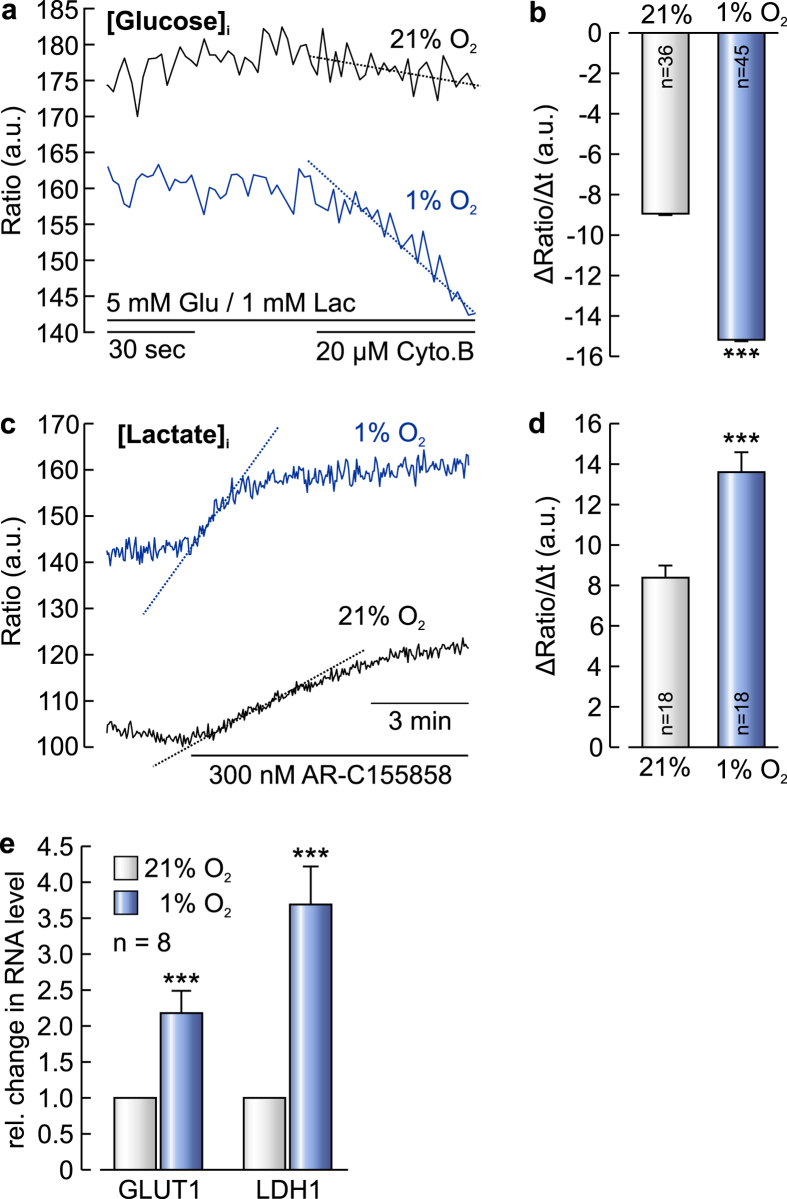
Glycolysis and lactate production are augmented under hypoxic conditions. (**a**) Relative change in intracellular glucose concentration in MCF-7 cells under normoxia (21% O_2_, black trace) and hypoxia (1% O_2_, blue trace), respectively, before and during inhibition of glucose uptake with 20 μM Cytochalasin B. (**b**) Rate of fall in intracellular glucose concentration, after inhibition of glucose uptake with Cytochalasin B in MCF-7 cells under normoxia (21% O_2_, light grey bar) and hypoxia (1% O_2_, blue bar), respectively. Hypoxia leads to a significant increase in glycolytic activity, as indicated by the increased rate of fall in glucose. (**c**) Relative change in intracellular lactate concentration in MCF-7 cells under normoxia (21% O_2_, black trace) and hypoxia (1% O_2_, blue trace), respectively, during inhibition of lactate efflux via MCT1 with 300 nm AR-C155858. (**d**) Rate of change in intracellular lactate concentration, after inhibition of lactate transport in MCF-7 cells under normoxia (21% O_2_, light grey bar) and hypoxia (1% O_2_, blue bar), respectively. Hypoxia leads to a robust increase in the rate of lactate production. (**e**) Relative change in the RNA level of GLUT1 and LDH1 in MCF-7 cells after three days under hypoxic conditions. Data are represented as mean ± SEM.

To investigate the significance of CAIX-mediated facilitation of lactate/H^+^ transport capacity for cancer cell metabolism and propagation, we determined changes in glycolysis and lactate production during hypoxia on the single cell level. We measured changes in intracellular glucose and lactate concentration in individual MCF-7 breast cancer cells, incubated three days under normoxic or hypoxic conditions, with *FLIP*^*12*^*glu-700* *μΔ6* and *Laconic*, respectively. Under hypoxic conditions, the glycolytic rate increased to 170%, as measured by the rate of decrease in intracellular glucose during inhibition of glucose import by 20 μM Cytochalasin B (Fig. 5a,b; ref. 34.)Lactate production increased to 162% under these conditions, as measured by the rate of increase in intracellular lactate during inhibition of lactate efflux with 300 nm AR-C155858 (Fig. 5c,d; ref. 35). Furthermore the mRNA levels of the glucose transporter GLUT1 and lactate dehydrogenase LDH-1, two markers for glycolytic metabolism, increased under hypoxic conditions (Fig. 5e).

### Non-catalytic augmentation of lactate efflux via CAIX facilitates cell proliferation

**Figure 6:**
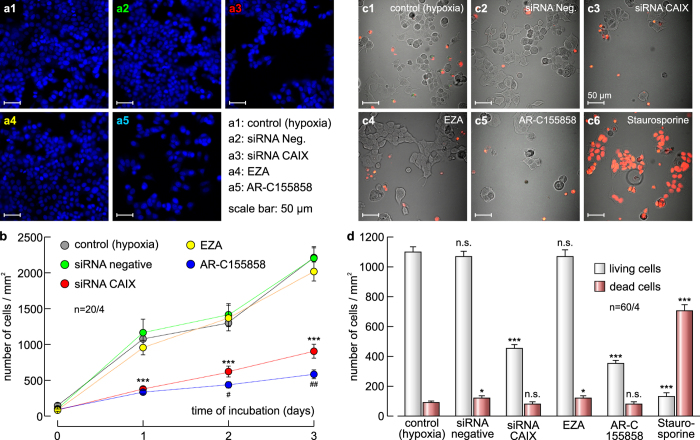
Knockdown of CAIX decreases cell proliferation. (**a**) Staining of nuclei with Hoechst (blue) in MCF-7 cells after 3 days in culture. Hypoxic cells remained either untreated (a1), mock-transfected with non-targeting negative control siRNA (a2), transfected with siRNA against CAIX (a3), incubated with the CA inhibitor EZA (a4), or incubated with the MCT1 inhibitor AR-C155858 (a5). (**b**) Total number of nuclei/mm^2^ in MCF-7 cell cultures, kept for 0–3 days under the conditions as described in (**a**). For every data point four dishes of cells were used and five pictures were taken from each dish at random locations, yielding 20 pictures/data point (n = 20/4). (**c**) Staining of dead MCF-7 cells with propidium iodide (red) after 3 days in culture. Living cells are visualised by phase contrast. Hypoxic cells remained either untreated (c1), mock-transfected with non-targeting negative control siRNA (c2), transfected with siRNA against CAIX (c3), incubated with the CA inhibitor EZA (c4), or incubated with the MCT1 inhibitor AR-C155858 (c5). As positive control, apoptosis was induced by application of staurosporine (c6). (**d**) Total number of living (grey) and dead (red) cells/mm^2^, kept for 3 days under the conditions as described in (**c**). For every data point 4 dishes of cells from two independent batches were used and 15 pictures were taken from each dish at random locations, yielding 60 pictures/data point (n =6 0/4). Data are represented as mean ± SEM.

The increase in glycolytic rate, as observed under hypoxic condition (see Fig. 5), leads to an increase in the production of lactate and protons, which have to be removed from the cell efficiently to suppress intracellular acidification, inhibition of metabolism and ultimately a decrease in cell proliferation. To investigate whether CAIX-mediated augmentation in lactate flux facilitates cancer cell proliferation under hypoxic conditions, we determined the number of MCF-7 cells kept under hypoxia at different conditions for three days (Fig. 6a,b). Indeed, knockdown of CAIX decreased cell proliferation to 27%–43% (as compared to cells transfected with non-targeting negative control siRNA), while transfection with non-targeting negative control siRNA had no significant effects on cell proliferation (102%–114%, as compared to untreated, hypoxic cells). Interestingly, total inhibition of lactate transport with 300 nM AR-C155858 decreased cell proliferation only slightly more than did knockdown of CAIX (24%–30%, as compared to untreated, hypoxic cells). Inhibition of CAIX catalytic activity with 30 μM EZA, however, had no effect on cell proliferation (94%–111% as compared to untreated, hypoxic cells). These results were confirmed on the triple negative breast cancer cell line MDA-MB-231 (Fig. S1g). Like in MCF-7 cells knockdown of CAIX decreased cell proliferation while transfection of the cells with non-targeting negative control siRNA and inhibition of CA catalytic activity with 30 μM EZA had no significant effect on cell proliferation. In contrast to MCF-7 cells inhibition of MCT1/2 transport activity with 300 nm AR-C155858 did not decrease cell proliferation. Since MDA-MB-231 cells primarily express MCT4 instead of MCT1 (Fig. S1d,e; ref. 37,36) a MCT1/2 inhibitor would not fully impair lactate efflux. However, incubation of MDA-MB-231 cells with the isoform-unspecific MCT inhibitor α-cyano-4-hydroxycinnamic acid (cinnamate, 1 mM) resulted in a severe reduction in cell proliferation (Fig. S1h), similar as did AR-C155858 in MCF-7 cells (see Fig. 6b).

To investigate whether the decrease in cell proliferation is due to an increase in cell death, we incubated MCF-7 cells under the same conditions as for the cell proliferation assay. After three days of incubation cells were stained with propidium iodide (PI) to identify dead cells and counted using a confocal laser scanning microscope with phase contrast illumination (Fig. 6c). While the number of living cells decreased after knockdown of CAIX and inhibition of MCT1 transport activity with AR-C155858, no significant increase in cell death could be observed under these conditions (Fig. 6d). Only when apoptosis was induced by addition of 50 μM staurosporine for 6–8 hours a significant increase in the number of dead cells could be observed. These data indicate that the observed decrease in the number of cells due to inhibition of MCT1 transport activity by knockdown of CAIX or application of AR-C155858 is not due to increased apoptosis but by a decrease in cell proliferation.

From these data it can be concluded that the non-catalytic augmentation of lactate/H^+^ efflux by CAIX can drive tumour cell proliferation under hypoxic conditions.

## Discussion

**Figure 7:**
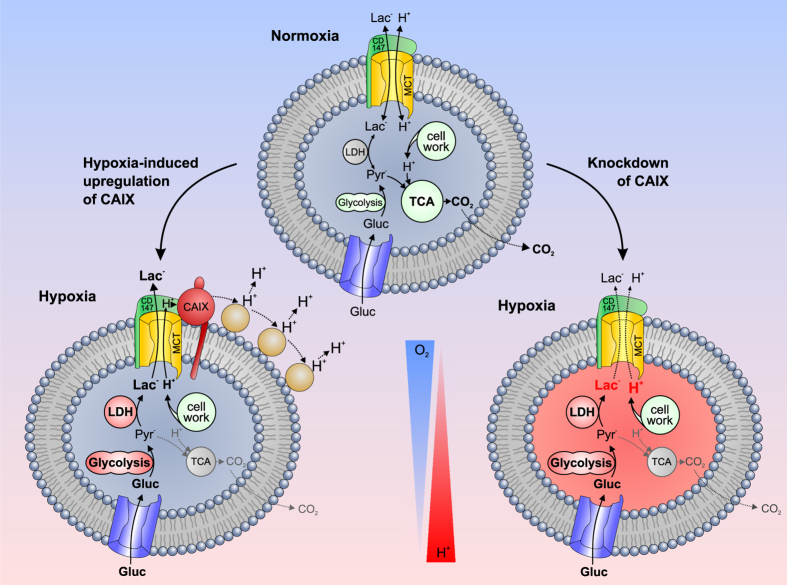
Schematic model of the CAIX-mediated increase in lactate transport in cancer cells under hypoxic conditions. Under normoxic conditions (upper scheme), cancer cells rely on glycolysis and oxidative energy production in the tricarboxylic acid cycle (TCA) to meet their metabolic requirements. Under hypoxic conditions, glycolysis becomes the prime energy source, which leads to vast production of lactate (produced from pyruvate by lactate dehydrogenase, LDH) and H^+^. Under these conditions (lower left scheme), hypoxia-regulated CAIX, which is directly bound to the complex of MCT and its chaperon CD147, could move protons between the transporter pore and extracellular protonatable residues (light brown circles). Thereby CAIX can function as a ‘H^+^-distributing antenna’ for the MCT to facilitate rapid extrusion of lactate and H^+^ from the cell. Knockdown of CAIX (lower right scheme) leads to loss of the ‘H^+^-distributing antenna’, which decreases MCT transport activity, leading to accumulation of lactate and H^+^ in the cytosol. A detailed description of the mechanism is given in the Discussion section.

The present study shows that lactate transport capacity in MCF-7 breast cancer cells is significantly increased under hypoxic conditions. The increase in lactate transport is mediated by a non-catalytic interaction between MCT1 and CAIX, expression of the latter is upregulated under hypoxia. Heterologous coexpression of MCT1 and CAIX in *Xenopus* oocytes identified the intramolecular H^+^-shuttle His200 in CAIX as a central component within this mechanism. Intracellular CAII, when directly bound to MCT1, can move protons between the transporter pore and surrounding protonatable residues at the plasma membrane, which dissipates local proton microdomains and facilitates H^+^/lactate cotransport when the proteins are heterologously expressed in *Xenopus* oocytes^33^,^38^. Like in the cytoplasm, diffusion of ions in the extracellular space (ECS) is restricted. It has been shown, that extracellular diffusion of ions is hindered by tortuosity of the ECS, proteoglycans at the outer membrane surface, charges at the extracellular matrix, and also by extracellular buffers^39^. Therefore an effective co-transport of lactate and H^+^ across the cell membrane requires efficient proton handling on the extracellular side of the plasma membrane. Like CAII, CAIX facilitates a histidine residue (H200) as an intramolecular H^+^ shuttle to move protons between its catalytic centre and the surrounding aqueous solution^40^. Therefore it appears plausible that CAIX, which is directly bound to the complex of MCT1 and its chaperon CD147 at the extracellular face of the plasma membrane, could move protons between the transporter pore and extracellular protonatable residues (Fig. 7).

This non-catalytic interaction between MCTs and hypoxia-regulated CAIX could be of crucial importance for cancer cell survival and progression within a hypoxic tumour environment: When an unrestricted supply of oxygen is available, the rate of O_2_ consumption and ATP production in many tumours is comparable to that found in the corresponding normal tissue^41^,^42^. However, oxygen delivery even within small solid tumours is often reduced by structural abnormalities in microvessels, disturbed microcirculation and deteriorating diffusion geometry^41^. As a result, most solid tumours develop hypoxic regions, in which cells have to undergo severe metabolic changes to survive. A direct consequence of this metabolic adaption is a glycolytic switch that leads to net increase in glucose consumption and lactate release. This increase in lactate release has been found to correlate with the aggressiveness of several types of human cancers^43^,^44^,^45^, which might reflect the metabolic adaptability of tumour cells to this extreme environment^12^. However, increased glycolysis does not have to necessarily depend on hypoxia. In ‘Warburg-phenotype’ tumour cells, which constitutively express HIF-1α, glycolysis is already increased under fully aerobic conditions^8^,^9^.

Under normoxic conditions MCF-7 breast cancer cells produce relatively low amounts of lactate as compared to other cancer cell lines^46^,^47^. However, hypoxia or anoxia induces a substantial increase in lactate production in this cell line (this study; Ref. 47). It has further been reported, that the exposure of the luminal-like MCF-7 cell line to a hypoxic environment promotes the onset of a more aggressive, basal-like breast carcinoma phenotype^48^ and triggers increased invasiveness^49^,^50^,^51^ and decreased sensitivity to different chemotherapeutics^52^,^53^. With these features, MCF-7 breast cancer cells seem to beautifully resemble the hypoxic switch observed in solid tumours.

It has been suggested that hypoxia induces upregulation of MCT4, but not MCT1, by a HIF-1α-dependent mechanism in different cell types, including cancer cells^11^,^54^. Since MCT4 does mainly mediate lactate efflux, it has been proposed that hypoxic cancer cells overexpress MCT4 to cope with the increase in H^+^/lactate production at low oxygen tension^4^,^12^. However, exposure of MCF-7 breast cancer cells to hypoxia increased lactate production, but did not result in an upregulation of MCT4 or MCT1 (this study). Interestingly, immunohistological studies on human breast cancer samples revealed that the most abundant MCT isoform in the plasma membrane was MCT1, not MCT4^55^. Furthermore expression of MCT1, but not MCT4, was significantly increased in breast carcinomas when compared to normal breast tissue and could be associated with poor prognostic variables such as basal-like subtype and high grade tumours^55^. From these data the authors concluded that MCT1 is the main isoform responsible for lactate plasma membrane transport in breast carcinoma^56^. The same group could also show that MCT1 (but not MCT4) is associated with CAIX in human breast cancer tissue, especially in more aggressive subtypes^57^. From this they concluded that the association between MCT1 and CAIX may result from hypoxia-mediated metabolic adaptations, which confer a glycolytic, acid-resistant, and more aggressive phenotype to cancer cells^57^.

In the present study we observed a significant increase in lactate transport capacity in MCF-7 cells under hypoxic conditions, which was not due to upregulation of MCT1 and MCT4, but was mediated by non-catalytic interaction between MCT1 and hypoxia-induced CAIX. Knockdown of CAIX decreased proliferation of MCF-7 cells, as did inhibition of MCT1 transport activity, while inhibition of CAIX catalytic activity had no effect on cell proliferation. A possible explanation for this observation is depicted in Fig. 7: Under normoxic conditions (upper scheme), cancer cells rely on glycolysis and oxidative energy production to meet their metabolic requirements (see 41,42). Under hypoxic conditions, glycolysis becomes the prime energy source, which leads to vast production of lactate and H^+^ (see^12^). However, in breast cancer cells hypoxia would not lead to an upregulation of MCT4 (see^55^,^56^), but to upregualtion of CAIX - which is associated with MCT1 (see^57^). CAIX would function as a ‘H^+^-distributing antenna’ for the MCT1 to facilitate rapid extrusion of lactate and H^+^ from the cell (lower left scheme). However, our studies on *Xenopus* oocytes have shown that CAIX also enhances transport activity of MCT4 by non-catalytic interaction. Therefore CAIX might well also increase lactate transport in MCT4-expressing tumour types under hypoxic conditions. Indeed knockdown of CAIX in hypoxic MDA-MB-231 cells -which primarily express MCT4 – also induced a decrease in cell proliferation as did inhibition of MCT transport activity with cinnamate. Knockdown of CAIX (lower right scheme) leads to loss of the ‘H^+^-distributing antenna’, which decreases MCT transport activity, leading to accumulation of lactate and H^+^ in the cytosol, which has been shown to impair cell proliferation and tumour growth^58^,^59^. Interfering with lactate release and pH regulation in cancer cells has therefore been suggested as a powerful tool in modern cancer therapy^3^,^14^ and different drugs that target activity of MCTs and CAIX are currently in clinical trial (for review see^27^). Indisulam, the current leading compound for inhibition of CAIX catalytic activity^12^,^60^ has already undergone Phase II clinical trial. However, no significant efficacy was observed for indisulam as a single agent in this trial on non-small cell lung cancer^61^. Another drug, AZD3965, which directly targets MCT1 transport activity is currently entering Phase I clinical trial^12^,^62^. Whether direct inhibition of MCT transport activity might interfere with lactate shuttling in healthy tissue like muscle or brain remains to be studied. The non-catalytic interaction between MCT1 and CAIX, observed in this study, may provide an additional target for specifically interfering with H^+^/lactate secretion in tumour tissue. Since coexpression of CAIX and MCT1 has not been shown in any healthy tissue, disruption of the CAIX H^+^-shuttle might specifically suppress MCT transport activity - and thereby H^+^/lactate secretion - in tumour tissue.

## Methods

### Cultivation of MCF-7 and MDA-MB-231 cells

The human breast adenocarcinoma cell lines MCF-7 and MDA-MB-231 were purchased from the German Collection of Microorganisms and Cell Cultures DSMZ, Braunschweig, Germany (DSMZ-No. ACC-115, ACC-732). MCF-7 cells were cultured in RPMI-1640 medium (Sigma-Aldrich, Schnelldorf, Germany), supplemented with 10% fetal bovine serum and 5 mM glucose, pH 7.2. MDA-MB-231 cells were cultured in Gibco Leibovitz-L15 medium (Life Technologies GmbH, Darmstadt, Germany), supplemented with 10% fetal calf serum, 5 mM glucose and 1% penicillin/streptomycin, pH 7.4. Both cell lines were incubated at 37 °C in 5% CO_2_, 21% O_2_/74% N_2_ (normoxia) or 1% O_2_/94% N_2_ (hypoxia) in humidified cell culture incubators.

### Inhibitors

CA catalytic activity was inhibited with 6-ethoxy-2-benzothiazolesulfonamide (EZA; 30 μM; Sigma-Aldrich). MCT1 was inhibited with AR-C155858 (300 nM; Tocris Bioscience, Bristol, UK). Isoform-unspecific inhibition of MCT activity was carried out with α-cyano-4-hydroxycinnamic acid (1 mM; Sigma-Aldrich). GLUT1 was inhibited with Cytochalasin B (20 μM, Sigma-Aldrich). With the exception of cinnamate, which was directly dissolved in the medium, all inhibitors were dissolved in dimethyl sulfoxide (DMSO) at a final DMSO concentration of max. 0.003%.

### Quantitative Real Time PCR

Total RNA was extracted from MCF-7 and MDA-MB-231 cells, respectively, using the RNeasy MinElute Cleanup Kit (Qiagen GmbH, Hilden, Germany). cDNA was synthesized with SuperScript III Reverse Transcriptase (Life Technologies) and random hexamer primers (200 ng; #SO142, Fisher Scientific GmbH, Schwerte, Germany). SYBR Green real time PCR was carried out with 13 μl SYBR Green Master mix (ABsolute QPCR SYBR Green Mix, Fisher Scientific), 10 nM primer and 15 ng cDNA in a final reaction volume of 20 μl. All samples were run in duplicate. RPL27 was used as a reference gene. The characteristics of the primers are shown in Table S1.

Thermal cycling was performed on a MyiQ™ real-time PCR system (Bio-Rad Laboratories GmbH, Munich, Germany) with the following protocol: 95 °C for 10 min, 45 cycles at 95 °C for 30 sec → 60 °C for 30 sec → 70 °C for 30 sec, and 72 °C for 5 min. To check for specificity of the primers the temperature was ramped up from 55 °C to 95 °C (at the rate of 0.5 °C/s). Melting curve analysis was performed according to the dissociation stage data and reactions with a single peak at the expected T_m_ were considered for further analysis. Calculation of the gene expression level was carried out with the program Rest 2005 (Corbett Research, Sydney, Australia and Technical University of Munich, Germany) using the comparative threshold method 2^−ΔΔCT^.

### pH Imaging in MCF-7 cells

Changes in intracellular pH were measured either with a confocal laser scanning microscope (LSM 700 with AxioExaminer.D1 upright microscope, Carl Zeiss Microscopy GmbH, Frankfurt, Germany) or an epifluorescence microscope (BX50WI upright microscope, Olympus Deutschland GmbH, Hamburg, Germany; with Polychrome IV epifluorescence unit, Till Photonics GmbH, Munich, Germany). For measurements on the confocal laser scanning microscope, cells were loaded with 10 μM of the acetoxymethyl ester of Seminaphthorhodafluor 5- and 6-carboxylic acid 5F (Invitrogen™ SNARF 5F-AM, Life Technologies) for 10–15 minutes. The scanning frequency was 0.4 Hz. SNARF was excited at 555 nm. The emitted light was separated with a variable dichroic mirror at 590 nm in a <590 nm and a >590 nm fraction. For ratiometric imaging the signals of the <590 nm fraction were divided by the signals of the >590 nm fraction. For measurements on the epifluorescence microscope, cells were loaded with 2 μM of the acetoxymethyl ester of 2’,7’-biscarboxyethyl-5,6-carboxy fluorescein (BCECF-AM; Life Technologies) for 10 minutes. BCECF was excited for 5 ms in an interval of 5 s, at a wavelength of 440 nm followed by 490 nm. The 535 nm fluorescence emission (F) of the two wavelengths’ was monitored through a 40x/0.8w objective with a peltier cooled CCD camera (Till Photonics). The emission strength at 490 nm is inverted proportional to pH while the emission strength at 440 nm remains constant during pH changes, which allows to achieve a pure pH-dependent signal by calculating the ratio F440/F490. Both systems were calibrated by the use of the K^+^/H^+^ exchanging ionophore nigericin (10 μM; Life Technologies) and the fluorescence signals converted to pH. Data analysis was carried out with the program Clampfit (Molecular Devices, Sunnyvale, California).

MCF-7cells were mounted under the microscope in petri dishes (Falcon™ Bacteriological Petri Dishes #351008, Fisher Scientific) and constantly perfused with medium at a rate of 2 ml/min at room temperature. The medium had the following composition: 143 mM NaCl, 5 mM KCl, 1 mM CaCl_2_, 1 mM MgSO_4_, 1 mM Na_2_HPO_4_, 10 mM 4-(2–hydroxyethyl)-1-piperazineethanesulfonic acid (HEPES), pH 7.2. In CO_2_/HCO_3_^−^- and lactate-containing media, respectively, NaCl was substituted by NaHCO_3_ or Na-lactate in equimolar amounts. To reproducibly measure the rate of lactate-induced acidification, cells were depleted of lactate for at least 15 minutes prior to lactate application.

### Lactate and glucose imaging in MCF-7 cells

MCF-7 cells were transduced with adenovirus carrying the lactate-sensitive FRET nanosensor *Laconic* (custom made by Vector Biolabs, Philadelphia, USA) or the glucose-sensitive FRET nanosensor *FLIP12glu-700* *μΔ6* (Vector Biolabs). Both FRET nanosensors have been described in detail previously (Laconic:^34^; FLIP12glu-700 μΔ6:^63^. For transduction, 1 × 10^4^ cells were resuspended in 800 μl of RPMI-1640 medium (supplemented with 10% FCS and 5 mM glucose) and plated on a petri dish (Falcon™ Bacteriological Petri Dishes #351008, Fisher Scientific). Immediately after plating either 0.3 μl of virus, containing Laconic (4.8 × 10^10^ PFU) or 1 μl of virus, containing FLIP12glu-700 μΔ6 (3.4 × 10^8^ PFU/ml) was added to the medium. Cells were incubated  overnight under normoxic conditions. Afterwards the transduction process was stopped by exchange of medium and cells were incubated for three days under normoxic and hypoxic conditions.

All imaging experiments were performed at room temperature with a Zeiss LSM 700 confocal laser scanning microscope as described for pH measurements in MCF-7 cells. *Laconic* and *FLIP12glu-700* *μΔ6*, respectively, were excited at 405 nm. The fluorescence was split at 508 nm into a <508 nm fraction and a >508 nm fraction. For ratiometric imaging of lactate concentration the <508 nm fraction was divided by the >508 nm fraction. For ratiometric imaging of glucose concentration the >508 nm fraction was divided by the <508 nm fraction. Data analysis was carried out with the program Clampfit (Molecular Devices).

### siRNA-mediated knock-down of carbonic anhydrase IX

CAIX was knocked down in MCF-7 and MDA-MB-231 cells, respectively, using siRNA (Ambion Silencer^®^ Select anti CA9 siRNA, s2270, Life Technologies). Non-targeting negative control siRNA (Ambion Silencer^®^ Select Negative Control No. 1 siRNA) was used as control. Cells were transfected with 50 pmol of siRNA in Lipofectamine RNAiMAX transfection reagent (Life Technologies). Transfected cells were incubated under hypoxic condition for 3 days. Knockdown efficiency was calculated by measurement of CAIX RNA and protein levels with real-time PCR and immunostaining, respectively.

### Immunocytochemistry in MCF-7 cells

Stainings were performed on MCF-7 cells cultivated in petri dishes for three days. Cells were fixed with 4% formaldehyde in phosphate-buffered saline (PBS) (Roti-Histofix^®^ 4%, Carl Roth GmbH + Co. KG) for 15 min. After blocking of unspecific binding sites with blocking solution (1:1000 Triton X-100, 1% goat serum, 3% bovine serum albumin (BSA)) for 60 min, cells were incubated with CAIX rabbit anti-human CAIX polyclonal antibody (diluted to 1:500; NB100-417, Novus Biologicals Ltd., UK) for 2 hours. Immunodetection of the primary antibody was carried out with an Alexa flour 488-linked secondary antibody (diluted to 1:1000; Alexa Fluor^®^ 488 Chicken Anti-Rabbit IgG (H + L) A-21441, Life Technologies). Nuclei were labelled with the fluorescent dye Hoechst (diluted to 1:1000; Life technologies). Images were taken with a confocal laser scanning microscope (Zeiss LSM 700). Quantification of the fluorescent signal was carried out with the software ImageJ (National Institutes of Health, USA).

### Measurement of cell proliferation

Cells were either treated with siRNA against CAIX (Ambion Silencer^®^ Select anti CA9 siRNA), non-targeting negative control siRNA (Ambion Silencer^®^ Select Negative Control No. 1 siRNA), the CA inhibitor EZA, the MCT1 inhibitor AR-C155858, or the isoform-unspecific MCT inhibitor α-cyano-4-hydroxycinnamic acid. Cells were plated at a density of ≥1 × 10^4^ cells/ml and incubated for 0–3 days under hypoxic conditions in a cell culture incubator. After incubation MCF-7 cells were fixed with Roti-Histofix^®^ 4% (Roth). Nuclei were stained with the fluorescent dye Hoechst (1:1000; Life technologies). For every condition four individual culture dishes were used. Five images were taken from each dish at random locations with a confocal laser scanning microscope (Zeiss LSM 700). The number of nuclei per image was counted using the software ImageJ. MDA-MB-231 cells were counted each day using a phase contrast microscope and placed back into the incubator after counting.

Dead MCF-7 cell were identified by staining with propidium iodide (PI). Cells were plated at a density of ≥1 × 10^4^ cells/ml and incubated for 3 days under the same conditions as described for the cell proliferation assay. As positive control, apoptosis was induced by addition of 50 μM staurosporine (6–8 hours before the measurement). After incubation 5 μl of 100 μg/ml of propidium iodide were added to each 500 μl of cell medium and cells were incubated for 30 min at room temperature. Images were taken by confocal laser scanning microscopy, combined with phase contrast illumination (Zeiss LSM 700). For each set of cells fifteen images from two sets of experiments were taken at random locations and the number of living cells (as evident in phase contrast) and dead cells (stained red by PI) per image were counted.

### Western blot analysis

For detection of CAIX rabbit anti-human CAIX polyclonal antibody (1:500; NB100-417, Novus Biologicals) was used. MCT1 was labelled with chicken anti-rat MCT1 polyclonal antibody (1:200; AB1286, Millipore). MCT4 was labelled with rabbit anti-rat MCT4 polyclonal antibody (1:250; AB3314P, Millipore). As a loading control, actin was labelled with mouse anti-human actin monoclonal antibody (1:1000; Anti-Actin (MO) Monoclonal LQ, MP69101, MP Biomedicals) or β-tubulin was labelled with anti-β-tubulin mouse monoclonal antibody (1:1000; Clone TUB 2.1 T5201, Sigma-Aldrich). Primary antibodies were labelled with goat anti-rabbit or goat anti-mouse IgG horseradish peroxidase-conjugated secondary antibody (1:1000; sc-2004 and sc-2005, Santa Cruz Biotechnology). Quantification of proteins was carried out with the software ImageJ. To allow comparison of different western blots, all measured protein concentrations on one blot were normalized to the concentration of one protein on the same blot.

### Single site mutation of CAIX

Mutation of histidine 200 to alanine in CAIX (CAIX-H200A) was carried out by PCR using a mix of Taq and Pfu polymerases (Fermentas High Fidelity PCR Enzyme Mix, Fisher Scientific) and modified primers, which contained the desired mutation (forward primer: 5′ - G CGC AAC AAT GGC **GC**A AGT GTG CAA CTG AC, reverse primer: 5′ - GT CAG TTG CAC ACT T**GC** GCC ATT GTT GCG C; exchanged nucleotides are labelled in bold). Human CAIX, cloned in the oocyte expression vector pGEM-He-Juel was used as template. The PCR was cleaned up using the Invitek MSB Spin PCRapace cleanup kit (Invitek GmbH, Berlin, Germany) and the template was digested with DpnI (Fermentas FastDigest DpnI, Fisher Scientific) before transformation into *E.coli* DH5α cells.

### Heterologous protein expression in *Xenopus* oocytes

Human CAIX was cloned into the oocyte expression vector pGEM-He-Juel, which contains the 5′ and the 3′ untranscribed regions of the *Xenopus* β-globin flanking the multiple cloning site. cDNA coding for rat MCT1, rat MCT2 and rat MCT4, respectively, cloned into the oocyte expression vector pGEM-He-Juel, was kindly provided by Dr. Stefan Bröer, Canberra^29^,^31^,^32^. Plasmid DNA was transcribed *in vitro* with T7 RNA-Polymerase (mMessage mMachine, Ambion Inc., Austin, USA) as described earlier^64^,^65^. *Xenopus laevis* females were purchased from Xenopus Express, Vernassal, France. Segments of ovarian lobules were surgically removed under sterile conditions from frogs anaesthetized with 1 g/l of 3-amino-benzoic acid ethylester (MS-222, Sigma-Aldrich), and rendered hypothermic. The procedure was approved by the Landesuntersuchungsamt Rheinland-Pfalz, Koblenz (23 177-07/A07-2-003 §6). As described earlier^64^,^65^, oocytes were singularized by collagenase (Collagenase A, Roche, Mannheim, Germany) treatment in Ca^2+^-free oocyte saline (pH 7.8) at 28 °C for 2 h. The singularized oocytes were left overnight in an incubator at 18 °C in Ca^2+^-containing oocyte saline (pH 7.8) to recover. Oocytes of the stages V and VI were injected with 5 ng of cRNA coding for MCT1 or MCT4, either together with 5 ng of cRNA coding for CAIX or alone. Measurements were carried out 3 to 6 days after injection of cRNA. CAIX was also directly injected as protein. For injection of protein, 20 ng of CAIX, isolated from secretion medium of CHO cells expressing recombinant proteins (kindly provided by Dr. William S. Sly, St. Louis), dissolved in 27.6 nl DEPC-H_2_O, were injected 12–24 h before electrophysiological measurements. Isolation of CA from secretion medium of CHO cells, as well as purification of the proteins with CA inhibitor columns has been described in detail previously^66^,^67^.

The oocyte saline had the following composition: 82.5 mM NaCl, 2.5 mM KCl, 1 mM CaCl_2_, 1 mM MgCl_2_, 1 mM Na_2_HPO_4_, 5 mM HEPES; titrated with NaOH to pH 7.0. In CO_2_/HCO_3_^−^- and lactate-containing saline, respectively, NaCl was substituted by NaHCO_3_ or Na-L-lactate in equimolar amounts.

### Measurement of intracellular H^+^ concentration in *Xenopus* oocytes

Changes in intracellular H^+^ concentration in oocytes were determined with ion-sensitive microelectrodes under voltage-clamp conditions. For measurement of intracellular H^+^ concentration and membrane potential, double-barrelled microelectrodes were used; the manufacture and application have been described in detail previously^65^,^68^. For two electrode voltage clamp a borosilicate glass capillary, 1.5 mm in diameter, was pulled to a micropipette and backfilled with 3 M KCl. This electrode was used for current injection and was connected to the head-stage of an Axoclamp 2B amplifier (Axon Instruments, USA). The actual membrane voltage was recorded by the reference barrel of the double-barrelled pH-sensitive microelectrode. Oocytes were clamped to a holding potential of −40 mV. As described previously^29^,^65^, optimal intracellular pH changes were detected when the ion-selective electrode was located near the inner surface of the plasma membrane. All experiments were carried out at room temperature.

### Determination of CA catalytic activity via mass spectrometry

Catalytic activity of CA in *Xenopus* oocytes was determined by monitoring the ^18^O depletion of doubly labelled ^13^C^18^O_2_ through several hydration and dehydration steps of CO_2_ and HCO_3_^−^ at 25 °C 69,70. The reaction sequence of ^18^O loss from ^13^C^18^O^18^O (m/z = 49) over the intermediate product ^13^C^18^O^16^O (m/z = 47) and the end product ^13^C^16^O^16^O (m/z = 45) was monitored with a quadropole mass spectrometer (OmniStar GSD 320; Pfeiffer Vacuum, Asslar, Germany). The relative ^18^O enrichment was calculated from the measured 45, 47, and 49 abundance as a function of time according to: log enrichment = log [49 × 100/(49 + 47 + 45)]. For the calculation of CA activity, the rate of ^18^O degradation was obtained from the linear slope of the log enrichment over the time, using the spreadsheet analysing software OriginPro 7 (OriginLab Corporation, Northampton, USA). The rate was compared with the corresponding rate of the non-catalysed reaction. Enzyme activity in units (U) was calculated from these two values as defined by Badger and Price^71^. From this definition, one unit corresponds to 100% stimulation of the non-catalysed ^18^O depletion of doubly labelled ^13^C^18^O_2_. Batches of 20 native oocytes or 20 oocytes expressing CAIX-WT or CAIX-H200A, were lysed in 80 μl oocyte saline, pipetted into the cuvette and the catalysed degradation was determined for 10 min.

### Calculation and statistics

Statistical values are presented as means ± standard error of the mean. For calculation of significance in differences, Student’s t-test or, if possible, a paired t-test was used. In the figures shown, a significance level of p ≤ 0.05 is marked with *, p ≤ 0.01 with ** and p ≤ 0.001 with ***.

## Additional Information

**How to cite this article**: Jamali, S. *et al.* Hypoxia-induced carbonic anhydrase IX facilitates lactate flux in human breast cancer cells by non-catalytic function. *Sci. Rep.* **5**, 13605; doi: 10.1038/srep13605 (2015).

## Supplementary Material

Supplementary Information
