# Supplementary material for: Hypoxia-induced carbonic anhydrase IX facilitates lactate flux in human breast cancer cells by non-catalytic function
Source: Sci Rep. 2015 Sep 4;5:13605. doi: 10.1038/srep13605 (PMC4559800; doi:10.1038/srep13605)
Supplement: Supplementary Information [file srep13605-s1.pdf]

Hypoxia-induced carbonic anhydrase IX facilitates lactate flux in human breast cancer cells by non-catalytic function

Somayeh Jamali[1], Michael Klier[1,2], Samantha Ames[1,2], L. Felipe Barros[3], Robert McKenna[4], Joachim W. Deitmer[2], and Holger M. Becker[1]

[1]Division of Zoology / Membrane Transport, FB Biologie, TU Kaiserslautern, P.O. Box 3049, D-67653 Kaiserslautern, Germany; [2]Division of General Zoology, FB Biologie, TU Kaiserslautern, P.O. Box 3049, D-67653 Kaiserslautern, Germany; [3]Centro de Estudios Científicos (CECs), Valdivia, Chile; [4]Department of Biochemistry and Molecular Biology, University of Florida, Gainesville, FL 32610, U.S.A.

Contact:
Holger M. Becker, Division of Zoology / Membrane Transport, FB Biologie, TU Kaiserslautern, P.O. Box 3049, D-67653 Kaiserslautern, Germany.

Content:

- **Supplementary Figure 1**: Determination of protein expression and cell proliferation in MDA-MB-231 cells
- **Supplementary Figure 2:** Hypoxia-induced increase in lactate/H^+ flux is independent of CAIX catalytic activity
- **Supplementary Figure 3:** CAIX enhances transport activity of MCT1 and MCT4 in *Xenopus* oocytes by non-catalytic interaction
- **Supplementary Table 1:** Characteristics of primers used for qRT-PCR

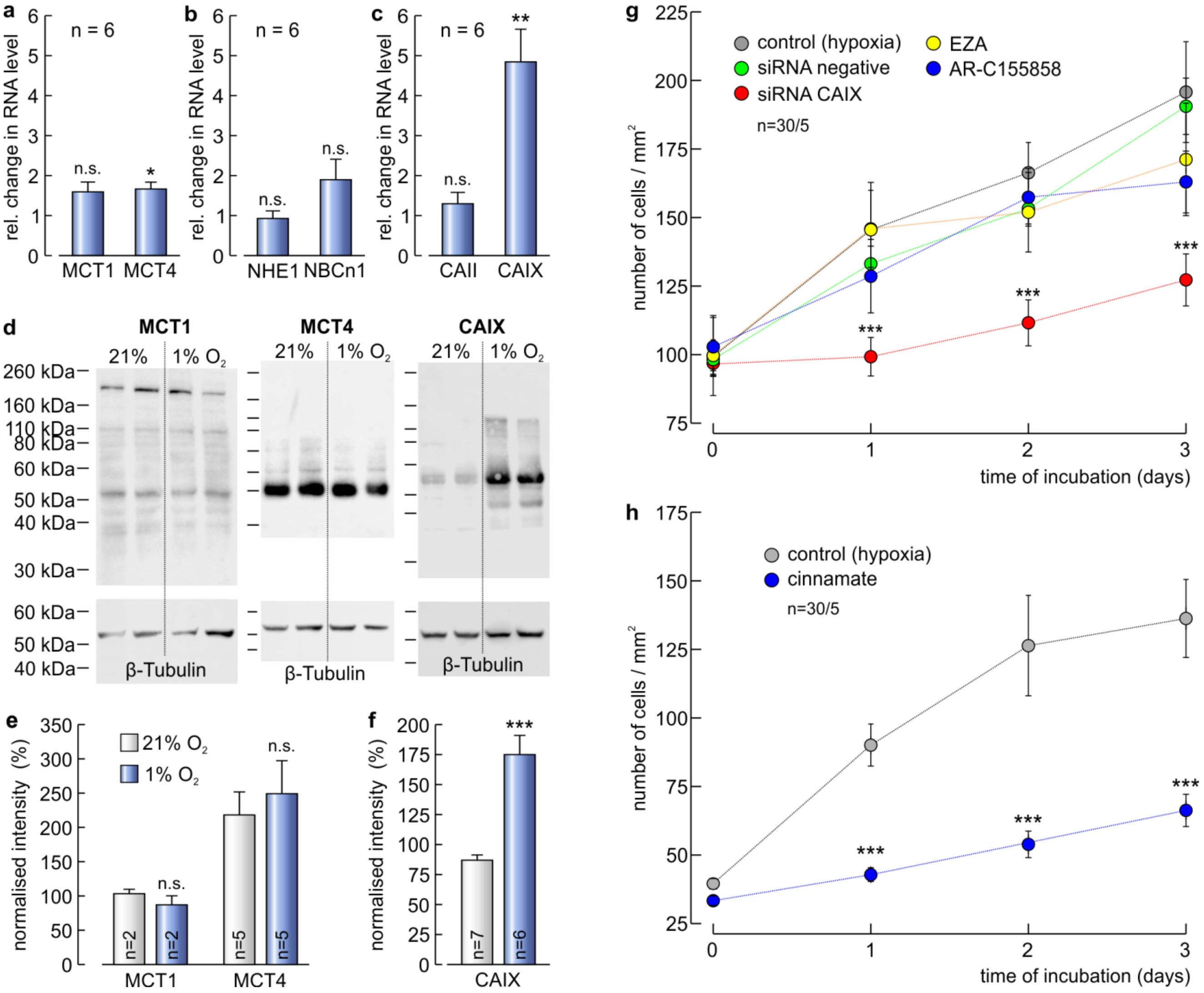

Supplementary Figure 1 | Determination of protein expression and cell proliferation in MDA-MB-231 cells. (a) Relative change in the RNA level of MCT1 and MCT4 in MDA-MB-231 cells after three days under hypoxic conditions. (b) Relative change in the RNA level of NHE1 and NBCn1 in MDA-MB-231 cells after three days under hypoxic conditions. (c) Relative change in the RNA level of CAII and CAIX in MDA-MB-231 cells after three days under hypoxic conditions. Expression level of CAIX is strongly upregulated, while the expression levels of MCT1, MCT4, NHE1 and NBCe1 show only slight changes. (d) Western blots of lysate from MDA-MB-231 cells, incubated under normoxic (21% O_2) and hypoxic (1% O_2) conditions, labelled for MCT1, MCT4, CAIX, and Actin, respectively. Quantification of the protein level of MCT1 and MCT4 by western blot analysis in MDA-MB-231 cells under normoxic (21% O_2, grey) and hypoxic (1% O_2, blue) conditions. (f) Quantification of

CAIX protein level by western blot analysis in MDA-MB-231 cells under normoxic (21% O_2, grey) and hypoxic (1% O_2, blue) conditions. (g) Total number of MDA-MB-231 cells/mm^2 after 1-3 days in culture. Hypoxic cells remained either untreated (grey), mock-transfected with non-targeting negative control siRNA (green), transfected with siRNA against CAIX (red), incubated with the CA inhibitor EZA (30 µM; yellow), or incubated with the MCT1 inhibitor AR-C155858 (300 nM; blue). Total number of MDA-MB-231 cells/mm^2 after 1-3 days in culture. Hypoxic cells remained either untreated (grey), or incubated with the isoform-unspecific MCT inhibitor cinnamate (1 mM; blue).

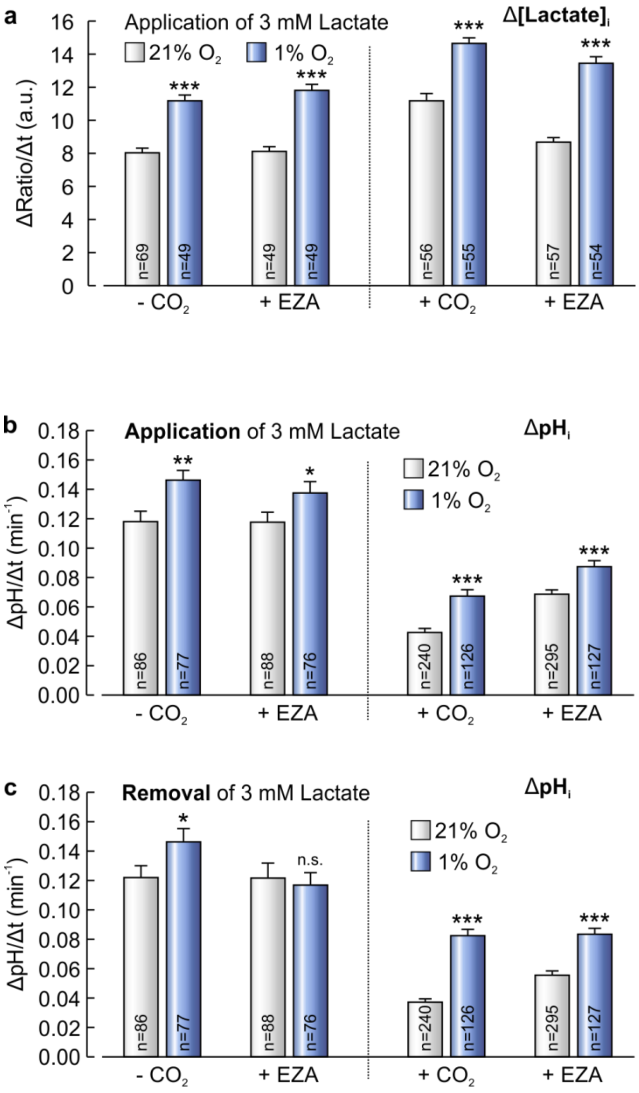

Supplementary Figure 2 | Hypoxia-induced increase in lactate/H$^+$ flux in MCF-7 cells is independent of CAIX catalytic activity. (a) Rate of change in intracellular lactate concentration in MCF-7 cells under normoxic (21% O_2) and hypoxic (1% O_2) conditions, respectively, as induced by application of 3 mM lactate in the absence and presence of 5% CO_2/15 mM HCO_3^- and 30 µM EZA, respectively. Hypoxia induces a significant increase in lactate flux both in the absence and in the presence of CO_2/HCO_3^- and EZA (blue bars). (b, c) Rate of change in pH_i in MCF-7 cells under normoxic (21% O_2) and hypoxic (1% O_2) conditions, respectively, as induced by application (b) and removal (c) of 3 mM lactate in the absence and presence of 5% CO_2/15 mM HCO_3^- and 30 µM EZA, respectively. Hypoxia induces a significant increase in the rate of proton flux, both in the absence and in the presence of CO2/HCO3- and EZA (blue bars). Data are represented as mean ± SEM.

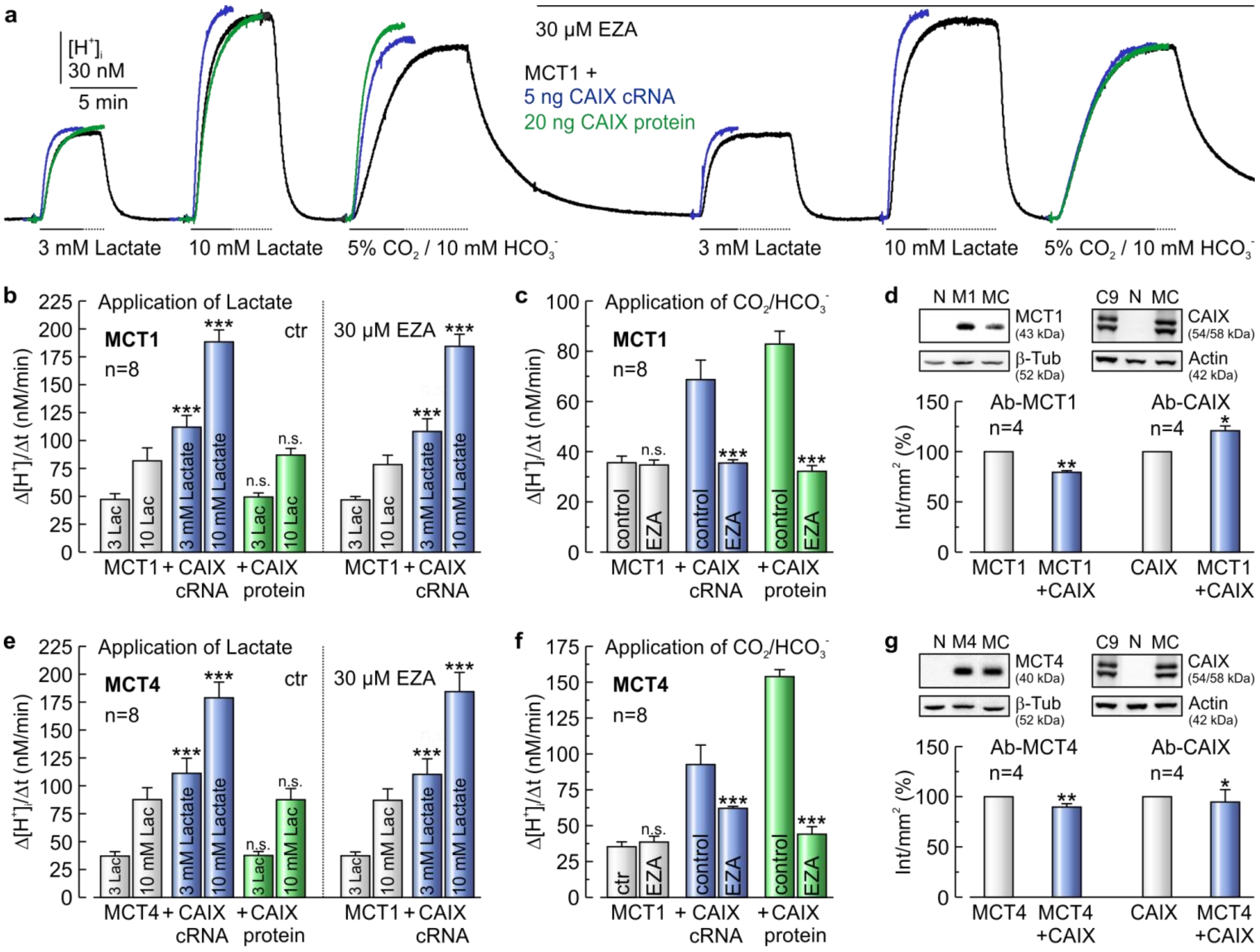

Supplementary Figure 3 | CAIX enhances transport activity of MCT1 and MCT4 in *Xenopus* oocytes by non-catalytic interaction. (a) Original recordings of intracellular H$^+$-concentration ([H$^+$]$_i$) in MCT1-expressing oocytes (black trace), injected with 5 ng of cRNA coding for CAIX (blue trace) or 20 ng of purified CAIX protein (green trace) during application of 3 and 10 mM lactate and 5% CO_2 / 10 mM HCO_3^-, in the absence and presence of 30 µM EZA. Rate of rise in [H$^+$]$_i$, as induced by application of lactate (b, e) or 5% CO_2 / 10 mM HCO_3^- (c, f) in MCT1- (b, c) and MCT4-expressing oocytes (e, f), injected with 5 ng of cRNA coding for CAIX or 20 ng of purified CAIX protein, in the absence and presence of 30 µM EZA. (d, g) Quantification of the protein levels of MCT1 (d), MCT4 (g) and CAIX by Western blot analysis. Gels were loaded with lysate of native (N), MCT1- (M1), MCT4- (M4)-, CAIX (C9) and MCT1/4+CAIX-coexpressing oocytes (MC), respectively. Actin and β-tubulin were used as loading control. Data are represented as mean ± SEM.

Supplementary Table 1 | Characteristics of primers used for qRT-PCR.

Gene	Primer	Seq 5’→ 3’	T_m (°C)	Amplicon size (bp)	Location
**CA II**	forward	AAACAAAGGGCAAGAGTGCTGACT	57.2	173	680-703
	reverse	TTTCAACACCTGCTCGCTGCTG	58.3		831-852
**CA IX**	forward	CTGGTGACTCTCGGCTACAGCT	57.82	153	1139-1160
	reverse	CTAGGATGTCACCAGCAGCCAGG	58.77		1269-1291
**Glut 1**	forward	TCTTCAGCCAGGGTCCACGTC	58.17	117	1706-1726
	reverse	CGTAGGGACCACACAGTTGCTC	57.28		1801-1822
**LDH-1**	forward	GGTGGTTGAGAGTGCTTATGAGGTG	57.36	116	971-995
	reverse	GGTGCACCCGCCTAAGATTCTTC	57.75		1064-1086
**HIF-1α**	forward	GCACTAGACAAAGTTCACCTGAGCCT	58.76	134	1918-1943
	reverse	TGGGTTCTTTGCTTCTGTGTCTTCAG	57.5		2026-2051
**NHE 1**	forward	CCTACGAGGAAGCCTGGAACCAG	58.46	144	2599-2621
	reverse	GTGGGTCTGAGCCGATGCG	57.68		2724-2742
**MCT 1**	forward	CATGATTGTTGGTGGCTGCTTGTC	57.28	108	627-650
	reverse	AGCCCAAGACCTCCAATGACTCC	58.05		712-734
**MCT 4**	forward	GCCATGCTCTACGGGACAGGT	58.3	98	371-391
	reverse	CCCAGCGACGCAAAGAGACC	58		449-468
**NBCn1**	forward	GGGTCAGCTTGTGGTCATCATGG	57.67	147	2502-2524
	reverse	GATTGTCGATCGCACCTTGGTAGG	57.7		2625-2648
**RPL27**	forward	GGTGGTTGCTGCCGAAATGGG	58.86	101	30-50
	reverse	TGTTCTTCACGATGACAGCTTTGCG	58.58		106-130
